# Bioaccumulation and Trophic Transfer of Heavy Metals in Marine Fish: Ecological and Ecosystem-Level Impacts

**DOI:** 10.3390/jox15020059

**Published:** 2025-04-18

**Authors:** Andra Oros

**Affiliations:** Chemical Oceanography and Marine Pollution Department, National Institute for Marine Research and Development (NIMRD) “Grigore Antipa”, 300 Mamaia Blvd., 900581 Constanta, Romania; aoros@alpha.rmri.ro

**Keywords:** heavy metals, marine fish, bioaccumulation, trophic transfer, ecotoxicology

## Abstract

Heavy metal contamination in marine ecosystems poses a critical environmental challenge, with significant implications for biodiversity, trophic dynamics, and human health. Marine fish are key bioindicators of heavy metal pollution because of their role in food webs and their capacity for bioaccumulation and trophic transfer. This review synthesizes current knowledge on the pathways and mechanisms of heavy metal accumulation in marine fish, focusing on factors that influence the uptake, retention, and tissue distribution. We explore the processes governing trophic transfer and biomagnification, highlighting species-specific accumulation patterns and the risks posed to apex predators, including humans. Additionally, we assess the ecological consequences of heavy metal contamination at population, community, and ecosystem levels, emphasizing its effects on fish reproduction, community structure, and trophic interactions. By integrating recent findings, this review highlights key knowledge gaps and suggests future research directions to improve environmental monitoring and risk assessment. Given the persistence and bioavailability of heavy metals in marine environments, effective pollution control strategies and sustainable fisheries management are imperative to mitigate long-term ecological and public health risks.

## 1. Introduction

Marine fish are essential components of marine ecosystems, playing a crucial role in maintaining biodiversity, regulating food webs, and supporting nutrient cycling [1]. In addition to their ecological importance, marine fish have significant economic value as a primary protein source for millions of people, especially in coastal communities where seafood supports livelihoods and local economies [2]. The fishing industry further contributes to global food security and economic development, reinforcing the need for the sustainable management of marine resources [3].

However, marine ecosystems face increasing threats from heavy metal pollution, which has emerged as one of the most persistent environmental challenges. Heavy metals (HMs) such as mercury (Hg), cadmium (Cd), and lead (Pb) are highly toxic and non-degradable, allowing them to persist in aquatic environments long after their initial introduction [4]. These metals enter marine ecosystems through natural processes—such as volcanic eruptions, hydrothermal vents, and rock weathering—and human activities, which contribute pollutants at much higher rates than natural sources [5,6,7].

Anthropogenic sources such as industrial emissions, metal smelting, petrochemical production, mining, and wastewater discharge release vast amounts of HMs into water bodies [8]. Agricultural activities also play a role, as fertilizers and pesticides containing metals like zinc (Zn), arsenic (As), and copper (Cu) wash into rivers and coastal areas, increasing contamination levels [9]. Additionally, fossil fuel combustion, especially from coal-fired power plants and vehicle emissions, releases airborne mercury and lead, which later settle into marine systems through atmospheric deposition [10].

Once introduced into marine environments, HMs follow complex pathways. Rivers carry pollutants from land to coastal areas, while atmospheric deposition spreads contaminants across wide oceanic regions [11]. Over time, metals accumulate in marine sediments, where they act as long-term reservoirs of contamination [12]. These sediments serve as both a sink and a secondary source, gradually releasing HMs back into the water column, where they can be absorbed by plankton, benthic organisms, and fish [13]. Through this interconnected system, HMs persist in marine food webs, even after external pollution inputs are reduced [14].

Marine fish are particularly vulnerable to heavy metal exposure through water, sediments, and diet [15,16,17]. Bioaccumulation causes metals to progressively build up in fish tissues, while trophic transfer increases the contamination risk as metals move up the food chain. This process ultimately affects apex predators, including humans, who consume contaminated seafood [18]. Heavy metal contamination disrupts marine biodiversity and poses major public health risks, particularly when fish species that are central to human diets accumulate hazardous concentrations of toxic metals [19]. Given these risks, continuous monitoring and mitigation strategies are essential to safeguarding both marine ecosystems and human health from the long-term impacts of heavy metal pollution [20].

This review examines the complex interactions between HMs and marine fish, with a primary focus on bioaccumulation mechanisms and trophic transfer processes. By exploring these pathways, it highlights the ecological consequences of heavy metal contamination, including disruptions at the population, community, and ecosystem levels, which can alter trophic dynamics and impact overall marine biodiversity. Due to the persistence of HMs in aquatic environments [21], their accumulation in fish not only affects ecosystem stability but also has implications for human health, as contaminated seafood remains a potential exposure route.

This review aims to synthesize the existing literature on heavy metal bioaccumulation and trophic transfer in marine fish, identify critical ecological impacts, and highlight knowledge gaps that warrant further investigation. By integrating findings across disciplines, it seeks to enhance the understanding of heavy metal pollution and its cascading effects on marine ecosystems and seafood safety. The insights provided can contribute to future research priorities, support risk assessment efforts, and inform sustainable management strategies to mitigate the long-term effects of heavy metal contamination [22].

## 2. Methodology

This review adheres to the PRISMA (Preferred Reporting Items for Systematic Reviews and Meta-Analyses) framework to ensure transparency, reproducibility, and methodological rigor. The PRISMA guidelines [23] provide a structured approach for literature selection and synthesis, thus minimizing the bias and enhancing the reliability. An initial literature search was conducted in the Web of Science (WoS) Core Collection for recent peer-reviewed articles published between 2015 and 2024, using the keywords “heavy metals” AND “marine fish” AND “bioaccumulation”. After applying the inclusion criteria, 141 relevant articles were selected. Studies were selected based on their explicit focus on the bioaccumulation and trophic transfer of heavy metals (HMs) in marine fish. Studies on freshwater or terrestrial environments, non-peer-reviewed sources, and articles without a clear focus on HMs or marine fish were excluded. The study selection process followed the following two-tiered approach: first, an initial screening of titles and abstracts to exclude irrelevant studies, followed by a full-text review to confirm adherence to inclusion criteria. Key details were extracted from each study, such as authors, publication year, journal, geographic region, fish species, types of heavy metals analyzed, assessment methods, and major findings. The extracted data were categorized into thematic areas such as bioaccumulation mechanisms and trophic transfer pathways. The most relevant and accessible studies, either open-access or institutionally available, are presented in tabular format, offering a comparative analysis of species-specific accumulation patterns and biomagnification dynamics.

Furthermore, a targeted search was conducted to explore topics related to the ecological effects of HM pollution, including impacts at the population, community, and ecosystem levels. To support this broader scope, we included recent open-access publications and full-text articles available through institutional subscriptions from leading academic publishers (e.g., Elsevier, Springer Nature, MDPI, Wiley, and Frontiers). This expanded the total number of reviewed studies to 235. The findings from this literature review have been incorporated into the relevant thematic discussions within the manuscript. Although this review focuses on ecological and trophic-level processes in marine fish, it does not cover molecular, biochemical, or cellular-level effects in detail. These are complex and fundamental components of HM toxicity that underline ecosystem-level responses but require separate, in-depth scientific analysis beyond the scope of the present study.

**Figure 1:**
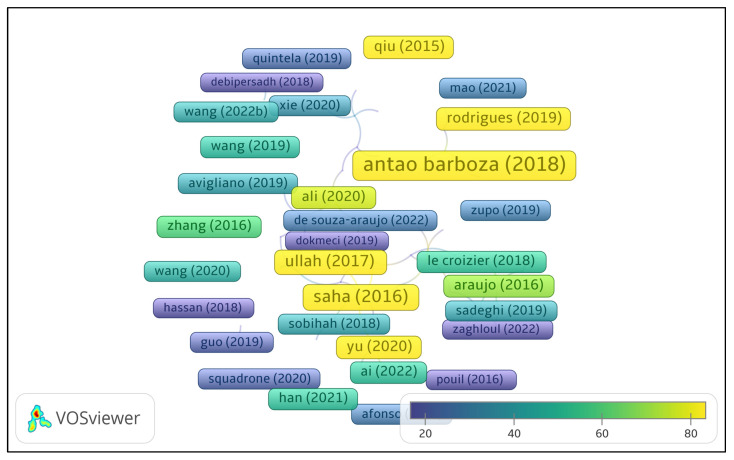
Citation network of the selected Web of Science records (minimum local citations ≥ 10) [18,26,27,28,29,30,31,32,33,34,35,36,37,38,39,40,41,42].

To analyze research trends, a bibliometric approach was applied using VOS viewer [24,25] on the selected Web of Science records, focusing on co-occurrence analysis (to identify key themes and emerging topics), citation frequency, and bibliographic coupling (to reveal research clusters based on shared references). Figure 1 presents a citation network based on Web of Science documents. Only publications that received at least 10 citations from other documents within the dataset are shown. Each node represents a document, with the node size reflecting the number of local citations. The color gradient corresponds to normalized citation counts, ranging from lower values (blue) to higher values (yellow), as shown in the legend. The spatial distribution of nodes indicates the similarity of their citation relationships. This map highlights the most locally influential documents within the selected literature set (Figure 1).

**Figure 2:**
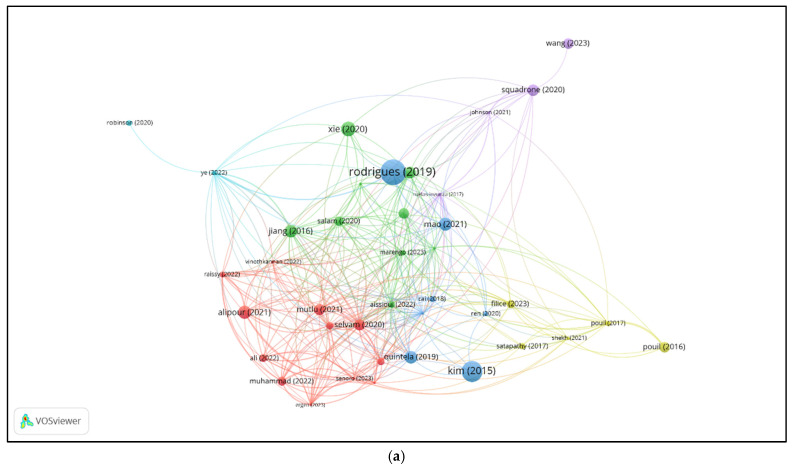

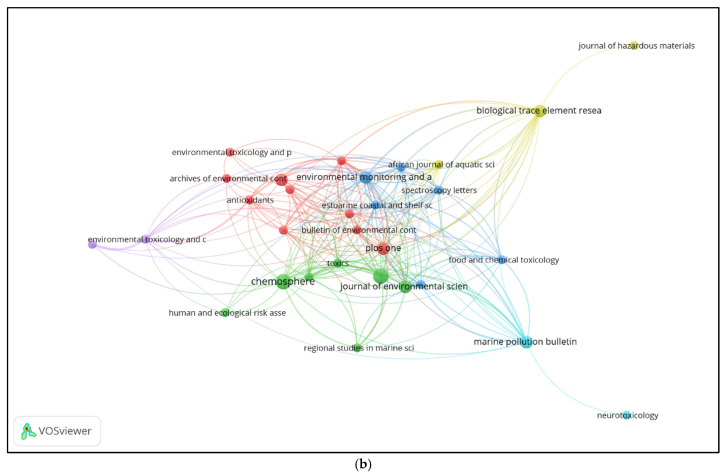
Bibliographic coupling analysis: documents [18,29,33,34,35,39,40,42,43,44,45,46,47,48,49] (**a**) and sources (**b**) of the selected Web of Science records.

Figure 2a displays the bibliographic coupling network of selected WoS documents. Each node represents a publication, with the node size indicating the strength of its connections to other documents based on shared references. A link between two nodes means the documents cite one or more of the same sources. The closer and more connected two documents are, the more references they have in common. Colors indicate clusters of publications with similar bibliographic profiles—groups of documents that cite overlapping sets of sources. These clusters suggest thematic similarity or alignment in research focus across the literature.

Figure 2b shows the bibliographic coupling network of sources, highlighting journals that share overlapping references. Journals that did not meet the set thresholds for the minimum number of publications and coupling strength were excluded from the visualization. Each node represents a journal, with the size indicating the number of publications in the dataset. Links between journals reflect the number of shared references cited by their articles. Journals are grouped into color-coded clusters, representing sources that cite similar literature and are thus thematically aligned. Central clusters include multidisciplinary environmental and marine science journals, while peripheral nodes indicate more specialized or distinct citation patterns. The map highlights the structural relationships among key publication venues based on shared bibliographic foundations.

## 3. Bioaccumulation of Heavy Metals in Marine Fish, Trophic Transfer, and Biomagnification

**Figure 3:**
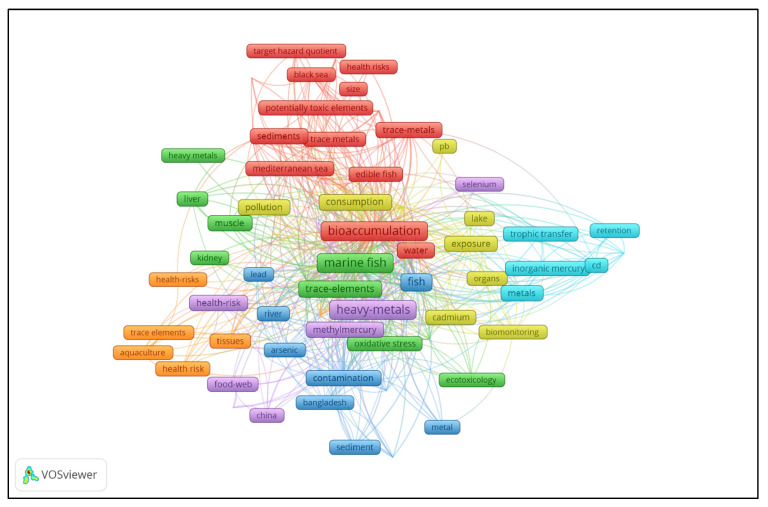
Co-occurrence network of keywords extracted from the Web of Science records.

Figure 3 presents the co-occurrence network of keywords e xtracted from the reviewed Web of Science records. Each node represents a keyword, with its size corresponding to its frequency of occurrence within the dataset. Connections between nodes indicate co-occurrence within the same documents, highlighting conceptual linkages. The different colors represent clusters of thematically related keywords, identified through VOS viewer’s clustering algorithm. These clusters reflect distinct research areas within the literature, including health risk assessment, environmental contamination, pollutant transfer, and bioaccumulation in marine organisms. The map offers a visual overview of major research topics and their interconnections, underscoring the interdisciplinary nature of the field, which spans environmental science, toxicology, and human health. The observed clustering patterns emphasize a strong focus on seafood safety and the ecological consequences of heavy metal contamination, particularly in relation to bioaccumulation in fish tissues and trophic transfer pathways. Emerging keywords such as oxidative stress and target hazard quotient highlight the growing interest in long-term ecological and human health impacts.

A summary of the reviewed studies addressing bioaccumulation and biomagnification mechanisms in marine fish is provided in Table 1 and Table 2.

Recent studies provide detailed insights into the mechanisms of heavy metal (HM) bioaccumulation in marine fish, emphasizing updated experimental and field-derived data. Fish accumulate metals through various routes, including gill uptake from water, dermal absorption, and ingestion of contaminated prey or sediments [50,51]. The extent of accumulation is influenced by the physicochemical characteristics of the metals and the physiological traits of the fish species [52].

Experiments confirm that gills, with their large surface area and ion-exchange functions, are key sites of HM uptake, especially for cadmium and mercury, which can bypass initial detoxification processes [26,53,54]. In benthic environments, dermal contact with metal-laden sediments significantly contributes to metal uptake, as field studies consistently report higher metal levels in demersal fish than in pelagic species [27,43,55,56,57].

Dietary exposure has also been documented as a dominant route for metal entry, especially for higher trophic-level species. Recent studies confirm that mercury biomagnifies through marine food chains, with top predators exhibiting higher tissue concentrations [28,44]. Seasonal influences have also been recorded, with higher bioaccumulation noted during dry periods when metal concentrations are less diluted [29].

At the cellular level, experimental analyses have shown that absorbed metals bind to proteins and lipids, forming persistent intracellular complexes. Cadmium and mercury, for instance, bind strongly to protein sulfhydryl groups, leading to prolonged tissue retention [5,30,56]. Methylmercury, being lipophilic, tends to accumulate in fatty tissues, particularly muscle [58]. These retention patterns are directly linked to metal toxicity and persistence.

While earlier studies often isolated single exposure routes, integrated field-based assessments now confirm that fish are concurrently exposed to metals via water, diet, and sediments [31,32,54,59]. Sediment exposure remains underexamined, even though isotope tracing shows that it plays a major role in the total metal burden [60]. Such multi-pathway exposure drives tissue-specific accumulation patterns, as demonstrated in recent demersal fish studies [27,43,56,61]. This complexity highlights the need for risk assessments that consider all relevant exposure pathways [20].

Recent biomonitoring has reinforced that HM distribution is highly tissue specific. Mercury, particularly in its methylated form, preferentially accumulates in muscle tissue due to its affinity for sulfur-containing amino acids [62]. This represents a significant concern for human consumers, particularly when consuming large predatory fish [27,33,45]. Cadmium, conversely, accumulates in the liver and kidneys, where it binds to metallothioneins, as evidenced in liver-targeted studies across different regions [54,63,64].

Other metals display distinct patterns. Lead preferentially binds to calcium-rich tissues such as bone and gill structures, affecting both skeletal and respiratory functions [26,54,65]. Essential metals such as zinc and iron are often found in both liver and muscle due to their involvement in enzymatic activity and metabolic processes [54,64]. Such patterns are crucial for assessing toxicity and dietary risks [66].

Environmental conditions, such as pH, temperature, and salinity, have also been identified as critical modulators of metal bioavailability [67,68]. Acidic conditions increase the solubility and uptake of lead and cadmium, while elevated temperatures accelerate respiratory rates and gill-mediated uptake [69,70].

Species-specific traits strongly influence how metals accumulate [71]. High-metabolism species (e.g., pelagic predators) tend to exhibit higher concentrations of bioaccumulated metals, reflecting both increased feeding rates and energetic demands [44]. In contrast, demersal fish display elevated HM levels due to prolonged contact with contaminated sediments [27,43,56]. Feeding strategies strongly modulate exposure: benthic feeders ingest sediment-bound metals, while pelagic and planktivorous fish are less exposed [27,32,43,56].

The trophic level has been shown to significantly influence bioaccumulation, particularly for mercury, which biomagnifies through the food web [28,45]. The detoxification capacity varies among species [72]. Those with greater metallothionein expression are more efficient at sequestering toxic metals like cadmium, thereby reducing free circulating concentrations [30,64]. Conversely, species with a limited detoxification ability bear higher tissue metal loads and are more vulnerable to toxic effects [61].

Together, these findings highlight the intricate interplay of environmental, physiological, and ecological factors governing HM bioaccumulation in marine fish [73]. Integrating these detailed insights into risk assessments is essential to evaluate ecosystem and human health risks from HM exposure.

**Table 1:** Summary of reviewed studies on bioaccumulation mechanisms and patterns in marine fish.

Reference	Region/Species/HM Studied	Key Findings
	Europe	
Kalantzi et al., 2015 [74]	Mediterranean Sea/European seabass (*Dicentrarchus labrax*) and gilthead seabream (*Sparus aurata*)/28 metals and trace elements	In farmed marine fish, metal accumulation varied by species and tissue, with seabass accumulating more elements than seabream. The liver and bone showed the highest concentrations, while muscle had the lowest. Feeding habits and environmental conditions, particularly oxygen levels, influenced bioaccumulation patterns. Fish in oxic environments accumulated more metals in muscle, bone, and intestine, while those in anoxic conditions had higher concentrations in liver and gills, reflecting sediment properties and metal bioavailability in aquaculture systems.
Pouil et al., 2016 [34]	Mediterranean Sea/turbot (*Scophthalmus maximus*)/Co, Mn, Zn	Metal assimilation in marine fish varied by diet, with fish prey contributing most to cobalt uptake, shrimp primarily influencing zinc accumulation, and manganese efficiently absorbed from both shrimp and ragworm. Crustaceans were the main source of essential metals, while polychaetes impacted seasonal manganese and cobalt intake. These findings highlight the significant role of diet composition in shaping trace metal bioaccumulation patterns.
Pouil et al., 2016 [35]	Mediterranean Sea/turbot (*Scophthalmus maximus*)/Cd, Co, Mn, Zn	Experimental validation of trophic metal transfer using radiolabeled food confirmed that a single-feeding approach effectively assessed the metal assimilation efficiency (AE). Manganese showed the highest AE (~23%), followed by cadmium and zinc (~13–14%), while cobalt had the lowest (~1%). Whole-body metal retention increased with multiple feedings, showing a linear accumulation pattern without storage limitations or significant changes in regulatory mechanisms. These findings highlight the reliability of experimental methods for trophic transfer and bioaccumulation studies, with implications for understanding the species-specific and metal-dependent assimilation efficiency in marine food webs.
Zupo et al., 2019 [27]	Mediterranean Sea/13 species of pelagic and demersal fish/Hg	Mercury bioaccumulation in marine fish is strongly influenced by habitat and life history traits. Demersal species accumulate more mercury due to sediment exposure, while pelagic fish, such as tuna, are exposed through migration across polluted areas. Mercury concentrates primarily in muscle tissue as methylmercury, with levels increasing with fish size and age—factors critical for accurate exposure and risk assessments.
Dokmecia et al., 2019 [31]	Marmara Sea/4 bottom fish species: sole (*Solea vulgaris*), whiting (*Merlangius merlangus*), striped red mullet (*Mullus surmuletus*), anglerfish (*Lophius piscatorius*)/Cd, Ni, Cu, Pb, Cr, Mn, Hg, Fe, As, Zn	Species-specific metal accumulation in marine fish was shaped by the habitat, trophic level, and feeding behavior. Essential metals like Fe, Zn, Cu, and Mn were abundant but metabolically regulated, while toxic metals such as Cd, Pb, Hg, and As showed species-dependent variation. Bottom-dwelling fish, including striped red mullet and sole, accumulated higher metal levels due to sediment exposure, whereas anglerfish and whiting had lower concentrations, likely reflecting differences in metabolism or environmental contact. These findings support the need for targeted contaminant monitoring across diverse marine species and habitats.
Vetsis et al., 2021 [75]	Thermaikos Gulf, Eastern Mediterranean Sea/28 demersal and pelagic fish species/27 metals	Heavy metal distribution in marine fish tissues revealed that gills, liver, and scales accumulated the highest element concentrations, while muscle showed the lowest. Pelagic species had higher metal levels than demersal fish. A positive correlation between the trophic level and metal concentrations in scales indicates the combined effects of dietary intake and environmental exposure. Due to their continuous growth and seawater contact, scales serve as effective indicators of long-term contamination, with implications for seafood safety and ecotoxicological risk assessment.
Mutlu, 2021 [43]	Eastern Black Sea/anchovy (*Engraulis encrasicolus*), Atlantic bonito (*Sarda sarda*), red mullet (*Mullus barbatus*), Mediterranean horse mackerel (*Trachurus mediterraneus*), whiting (*Merlangius merlangus*)/Al, Cd, Cr, Cu, Fe, Mn, Pb, Zn	Metal accumulation in marine fish varies by species, with feeding behavior, habitat, and physiology playing key roles. Anchovy accumulated the highest levels of aluminum, cadmium, chromium, and manganese due to its plankton-based diet and filtration rates. Mediterranean horse mackerel showed the highest lead and zinc concentrations, reflecting its benthopelagic feeding habits, while Atlantic bonito accumulated the most iron and copper, linked to its active predatory lifestyle and higher metabolic demands. These patterns underscore the importance of ecological factors in shaping metal bioaccumulation.
Filice et al., 2023 [44]	Northwestern Mediterranean Sea/shark (*Galeus melastomus*), European conger (*Conger conger*), red mullet (*Mullus barbatus*), common dentex (*Dentex dentex*), John Dory (*Zeus faber*), gilthead seabream (*Sparus aurata*)/Hg, Pb, Cd, Zn, Cu, Ni, Fe	In the top predator *Galeus melastomus*, mercury and lead biomagnified, while cadmium and copper were more prevalent in benthic species, reflecting habitat-driven accumulation. Liver and gill tissues showed the highest bioaccumulation, with muscle concentrations remaining lower. Metal exposure induced oxidative stress, with increased antioxidant enzyme activity in species with higher metal loads. Liver and gills experienced the most oxidative damage, reinforcing their detoxification role. These findings highlight the influence of feeding behavior and habitat on bioaccumulation patterns, and identify oxidative stress as a critical biomarker for metal exposure, with implications for pollution assessment and fisheries management.
Beauvieux et al., 2024 [61]	Gulf of Lions (NW Mediterranean Sea)/gilthead seabream (*Sparus aurata*)/Al, As, Be, Bi, Cd, Cr, Cu, Hg, Li, Ni, Pb, Rb, Sr, Ti, Tl, Zn	In juvenile seabream across nursery lagoons, trace element accumulation varied by individual and habitat, with muscle showing multiple inorganic pollutants. Size-related trends revealed aluminum increasing with fish size, while arsenic, lithium, strontium, thallium, and zinc decreased, highlighting habitat-specific exposure risks. Proteomic analysis indicated that pollutant exposure disrupted liver cellular organization and protein transport, potentially reducing lifespan and increasing disease susceptibility. Red muscle showed increased metabolic activity, suggesting energy trade-offs that affect growth and reproduction. These biomarkers provide a molecular fingerprint of pollution effects, supporting long-term ecosystem health monitoring.
	Asia	
Qiu, 2015 [26]	Daya Bay, South China/planktivorous fish (*Stromateoides argenteus*), carnivorous fish (*Saurida undosquamis*)/Cu, Zn, Pb, Cd	Tissue-specific metal accumulation patterns in marine fish reveal functional roles and exposure pathways: copper and zinc concentrated in muscle due to their strong protein-binding affinity, lead accumulated in the liver as part of detoxification, cadmium was stored in the kidneys, and gills reflected environmental exposure. These patterns underscore the importance of assessing multiple organs for accurate health risk evaluation and contaminant monitoring.
Guo et al., 2019 [32]	China, Guangdong/yellowstripe goby (*Mugilogobius chulae*)/Cd	Cadmium bioaccumulation in demersal fish occurs through multiple pathways—water, sediment, and diet—with tissue-specific uptake patterns. Gills primarily absorb Cd from water, while the gastrointestinal tract handles dietary and sediment-derived uptake. Muscle and carcass accumulate Cd from both diet and water sources. Triple stable isotope tracing revealed the complexity of these pathways, offering insights into metal transfer, ecological risk, and human health implications in marine food webs.
Ren et al., 2020 [46]	Yellow Sea (China)/flounder (*Paralichthys olivaceus*)/methylmercury (MeHg)	Methylmercury (MeHg) bioaccumulation in marine fish was tissue-specific, with the highest concentrations in the liver, followed by gills and muscle. Accumulation increased with exposure dose, and the liver played a key role in detoxification, while muscle tissue retained lower concentrations due to growth dilution effects. These findings highlight the liver’s critical detoxification function and underscore the importance of tissue-specific assessments for seafood safety.
Xie et al., 2020 [29]	Pearl River Estuary (China)/7 demersal species (eel goby, tonguesole, sillago, catfish, bombay duck, belanger croaker, mullet); 3 pelagic species (pomfret, anchovy, shad)/Cr, Mn, Cu, Zn, As, Se, Cd, Hg, Pb	Metal accumulation in marine fish varied by species, with eel gobies accumulating the highest levels of copper, lead, mercury, manganese, and selenium, tonguesole showing the most inorganic arsenic, and catfish retaining the highest zinc concentrations. Non-essential metals like arsenic and lead were significantly higher during the dry season, likely due to dilution effects in the wet season, indicating seasonal variations in exposure. These findings emphasize the role of ecological and physiological traits in shaping species-specific metal burdens.
Selvam et al., 2020 [47]	Gulf of Mannar, Tamil Nadu, India/multiple edible marine fish species/Hg, Pb, Cd, Cu, Zn, Fe, Ni, Cr	Benthic species accumulated higher metal concentrations than pelagic species, driven by feeding habits, habitat, and metabolism. Liver and gills exhibited the highest metal levels, reflecting uptake from both diet and water. High metal burdens may compromise the liver’s detoxification function. These findings emphasize the importance of species-specific bioaccumulation patterns for environmental risk assessments and highlight the physiological effects of metal accumulation on key tissues.
Kumar et al., 2021 [76]	Mumbai Coast, India/30 marine fish species from various fishing harbors/Cr, Mn, Co, Cu, Zn, Se, As, Sr, Cd, Sn, Sb, Pb	Heavy metal and metalloid bioaccumulation in marine fish varied by species, tissue, and feeding behavior. Zinc, chromium, and strontium were most abundant in muscle, while lead and cadmium levels remained low. Carnivorous and benthic species accumulated more metals than herbivores. Tissue-specific trends revealed Zn and Cr concentrated in muscle, Mn and Cu in the liver, and lower accumulation in gills. These patterns reflect distinct exposure pathways and are valuable for monitoring coastal pollution.
Huang et al., 2022 [56]	East China Sea/ 9 species (*Johnius belengerii*, *Chrysochir aureus*, *Collichthys lucidus*, *Muraenesox cinereus*, *Sebastiscus marmoratus*, *Coilia macrognathos*)/As, Hg, Cd, Cu, Zn, Pb, Cr	Bioaccumulation patterns in marine fish are shaped by habitat and feeding strategies. Demersal species exhibited higher levels of zinc and copper due to prolonged sediment exposure, while pelagic and midwater species had lower concentrations. Zinc and copper were the most abundant metals overall, with the liver showing the highest levels due to its detoxification role, followed by moderate concentrations in muscle and intermediate levels in gills. These findings underscore the liver’s utility as a bioindicator, muscle tissue’s importance for dietary exposure, and the generally lower biomagnification potential of toxic metals like Pb and Cd.
Al Solami, 2022 [77]	Central Red Sea/5 coral reef-associated species (*Lethrinus mahsena*, *Acanthurus gahhm*, *Siganus rivulatus*, *Scarus ferrugineus*, *Hipposcarus harid*)/Cu, Pb, Mn	Bioaccumulation patterns in coral reef-associated fish varied by species, driven by feeding behavior and habitat. Herbivorous species generally exhibited lower lead levels compared to carnivorous and omnivorous fish, reflecting differences in the trophic position and environmental exposure. These findings highlight the role of diet and habitat in shaping heavy metal accumulation in reef ecosystems.
Javanshir Khoei, A. (2022) [57]	Oman Sea/ *Scomberoides commersonnianus*, *Rastrelliger kanagurta*, *Saurida tumbil*, *Parastromateus niger*, *Alepes melanoptera*, *Nemipterus japonicus*, *Psettodes erumei*/Ni, Cr, Hg, Cd, Pb	In commercially important marine fish, demersal species accumulate higher levels of heavy metals than pelagic species, with accumulation rates differing across species. Mercury, lead, and cadmium concentrations correlated with fish size in *Psettodes erumei* and *Parastromateus niger*, indicating cumulative exposure over time. Bioaccumulation patterns were shaped by species-specific feeding behavior, metabolism, and habitat use, with important implications for seafood safety and trophic transfer assessments.
Yang et al., 2022 [69]	South China Sea/84 fish species collected from nearshore and offshore regions/Ni, Cu, As, Cd, Pb, Hg	In marine environments, nearshore waters showed elevated copper levels due to anthropogenic inputs, while offshore fish accumulated more heavy metals despite lower direct pollution. This suggests that bioaccumulation is influenced more by ecological factors—such as feeding habits and food web dynamics—than by water contamination alone. These findings emphasize the importance of integrating both environmental and biological variables in heavy metal risk assessments.
Perumal et al., 2023 [78]	Southeast Coast, Tamil Nadu, India/13 marine fish species/Fe, Mg, Zn, Cu, Cd, Pb, Hg	In commercially important tropical fish, metal accumulation varied by the species and landing site, with the general trend Mg > Fe > Zn > Cu > Hg > Pb > Cd. Muscle tissue was the primary site of accumulation, shaped by species-specific traits such as feeding behavior, swimming activity, and genetic factors. These patterns reflect both spatial contamination differences and environmental influences on bioaccumulation.
	Africa	
Le Croizier et al., 2016 [54]	Dakar and Casamance, Senegal/*Scomber japonicus*, *Diplodus bellottii*, *Caranx rhonchus*, *Chloroscombrus chrysurus*, *Galeoides decadactylus*)/Cd, Pb, Fe, Zn, As, Ni, Sn, Co	Regional pollution strongly influences metal bioaccumulation in marine fish. Specimens from Dakar showed higher concentrations of Cd, Pb, and Fe—particularly in benthic feeders—due to industrial activity and sediment-based diets, while fish from less-impacted Casamance exhibited lower levels. The liver was the main site for toxic metal accumulation, muscle tissue retained essential metals relevant to human exposure, and gills reflected environmental contact. Fatty acid and trophic markers further linked contamination to dietary sources, emphasizing the roles of habitat and feeding behavior in shaping bioaccumulation patterns.
Le Croizier et al., 2018 [30]	Canary Current Large Marine Ecosystem (CCLME)/*Dicentrarchus labrax* (European sea bass), *Solea senegalensis* (Senegalese sole)/cadmium (Cd)	Species-specific differences in cadmium bioaccumulation and detoxification were observed under controlled dietary exposure. Sea bass absorbed Cd more rapidly in liver and muscle than sole and exhibited higher biliary excretion efficiency. While no metallothionein (MT) induction was detected, higher basal MT levels in sea bass likely supported detoxification. Hepatic Cd transfer to muscle was greater in sea bass, though both species retained Cd in muscle over time. These findings highlight organ-specific metal distribution and raise questions about the reliability of MTs as biomarkers.
Debipersadh et al., 2018 [36]	Durban Coast, South Africa/*Trachurus capensis* (Cape horse mackerel)/Al, As, Pb, Cr, Mn, Zn, Cu, Ba	In a commercially important fish species, lead (Pb) showed the highest tissue concentrations, followed by zinc (Zn), with manganese (Mn) accumulating notably in gills. Pb was more concentrated than arsenic, chromium, and Mn, and exhibited widespread distribution across all tissues. Zn was also consistently present at notable levels. No significant variation was found in gill metal accumulation. These patterns are important for evaluating seafood safety risks and guiding fisheries management.
Debipersadh et al., 2018 [37]	South Durban Basin, South Africa/6 edible fish species: *Epinephelus andersoni*, *Chrysoblephus puniceus*, *Cheimerius nufar*, *Pachymetopon grande*, *Trachurus trachurus*, *Scomber colias*/Al, As, Cd, Cr, Cu, Mn, Pb, Zn	Toxic metal accumulation in edible marine fish varied by species and tissue. Horse mackerel had the highest copper and chromium levels, slinger seabream showed elevated lead, and bronze seabream accumulated the most manganese. The liver was the main site of overall metal storage, manganese concentrated in gills, and zinc was most abundant in muscle tissue. These findings highlight species- and organ-specific bioaccumulation patterns, underscoring the need for targeted monitoring to support exposure risk assessment.
Kaddour et al., 2023 [64]	Oran Bay, Algeria/European hake (*Merluccius merluccius*)/Cd, Pb, Zn	Bioaccumulation in demersal hake is influenced by biological factors such as sex and habitat exposure. Zinc was the most abundant and bioavailable metal, with females showing higher Zn and Cd levels in the liver, while males had greater Pb accumulation. The liver served as the primary site for metal storage—especially Zn—due to its detoxification function, whereas muscle tissue retained lower but still relevant Zn concentrations linked to dietary intake. While Zn plays essential physiological roles, Cd and Pb, though less prevalent, pose toxicity risks due to their accumulation in critical tissues.
	America	
Ruelas-Inzunza et al., 2017 [79]	Pacific coast, SE Gulf of California/white mullet (*Mugil curema*), striped mullet (*Mugil cephalus*)/total mercury (THg) and methylmercury (MeHg)	Mercury accumulated more in the liver than in muscle for both mullet species. In *M. curema*, mercury levels in both muscle and liver correlated with fish weight, while, in *M. cephalus*, only liver mercury levels were linked to fish size. These findings suggest that mercury bioaccumulation is influenced by species-specific metabolic factors, highlighting the importance of tissue-specific patterns and size in bioaccumulation assessments.
Avigliano et al., 2019 [38]	La Plata Basin (South America)/streaked prochilod (*Prochilodus lineatus*)/Ag, As, Cd, Cu, Cr, Hg, Ni, Pb, Se, U, V, Zn	In this species, the liver was the primary storage site for metals, concentrating silver, copper, mercury, and zinc, while muscle tissues showed lower metal levels. Non-muscle tissues exhibited selective metal retention, and the absence of a direct correlation between sediment/water and tissue concentrations suggests that migratory behavior significantly influences metal exposure and retention. These findings highlight the importance of tissue-specific accumulation and migration in bioaccumulation patterns, with implications for ecological risk assessment and fisheries management.
Squadrone et al., 2020 [33]	Caribbean Sea/Indo-Pacific lionfish (*Pterois* spp.)/23 trace elements, 16 rare earth elements	Bioaccumulation of trace and rare earth elements in marine fish was highest in the liver and kidneys, reflecting their detoxification roles, while muscle tissue accumulated significantly lower metal levels. Cadmium and lead concentrations remain within regulatory safety limits for human consumption, confirming muscle as a safer dietary source. These findings emphasize organ-specific metal accumulation patterns and their implications for human health risk assessment.
Johnson et al., 2021 [45]	Atlantic Coast (Florida, USA)/invasive lionfish (*Pterois volitans/miles*)/total mercury (THg)	Mercury concentrations in lionfish increased with fish size and varied by location and sex, with the highest levels found in muscle, followed by the liver and adipose tissue. Despite this, mercury levels were lower than in other predatory reef fish, placing lionfish in Florida’s least restrictive consumption advisory category. These findings highlight the importance of size- and tissue-specific mercury accumulation in risk assessments for human consumption.
Shekh et al., 2021 [48]	North America/rainbow trout (*Oncorhynchus mykiss*), white sturgeon (*Acipenser transmontanus*)/cadmium (Cd), copper (Cu)	White sturgeons showed greater resistance to cadmium (Cd) than rainbow trout, effectively detoxifying Cd by storing it in the Biologically Inactive Metal Pool (BIM), which includes heat-stable proteins and metal-rich granules. In contrast, trout accumulated more Cd in the Biologically Active Metal Pool (BAM), leading to cell damage. For copper (Cu), sturgeons were more sensitive than trout, though no significant differences in BAM/BIM levels were observed, suggesting that other physiological mechanisms contribute to Cu sensitivity in sturgeon. These findings provide valuable insights into species-specific metal sensitivity and detoxification mechanisms, with implications for environmental risk assessment and water quality guidelines for Cd and Cu contamination.
Serviere-Zaragoza et al., 2021 [80]	Pacific coast, Gulf of California (Santa Rosalía and Bahía de La Paz)/*Kyphosus vaigiensis* (herbivore), *Stegastes rectifraenum* (omnivore), *Balistes polylepis* (carnivore)/Cd, Pb, Cu, Zn, Fe	Metal concentrations varied significantly between species, locations, and seasons. The omnivorous *S. rectifraenum* showed the highest levels of Cd and Pb, *B. polylepis* (carnivore) had elevated Cu and Zn, while *K. vaigiensis* (herbivore) accumulated more Fe. These patterns were linked to species-specific feeding habits and exposure. Although differences were sometimes significant between sites and seasons, the estimated metal concentrations in muscle did not exceed safety thresholds for human consumption.
Figueiredo et al., 2020 [81]	Pacific coast, Gulf of California (Guaymas Basin)/*Triphoturus mexicanus*, *Benthosema panamense* (mesopelagic, diel vertical migrators)/Cr, Mn, Co, Ni, Cu, Zn, As, Se, Cd, Pb	Both species accumulated Zn and As at the highest levels, with *T. mexicanus* exhibiting significantly higher Cr, Cu, Zn, and Pb than *B. panamense*. The interspecific differences were attributed to physiological and behavioral traits rather than environmental availability. *T. mexicanus*, being more sedentary and dwelling deeper during the day, showed greater trace element accumulation.
Souza-Araujo et al., 2022 [28]	Amazon Coast/47 fish species, including cartilaginous and teleost fish/As, Cd, Hg, Pb	On the Amazon Coast, reef-associated fish exhibited over twice the mercury concentrations of demersal species, driven by dietary differences and environmental factors—such as organic matter input and low oxygen levels—that enhance mercury methylation and bioavailability. This led to greater accumulation in top predators through biomagnification. The findings underscore the influence of habitat conditions on metal distribution and highlight significant risks to seafood safety and human health, supporting the need for targeted mercury monitoring in reef ecosystems.
	Antarctica	
Queiros et al., 2023 [82]	Southern Ocean/Antarctic toothfish (*Dissostichus mawsoni*)/27 trace elements	In an Antarctic top predator, essential elements like potassium, sodium, phosphorus, calcium, and magnesium were most abundant, while rare earth elements had the lowest concentrations. Elemental levels varied spatially, reflecting regional differences in water chemistry and diet. Larger fish exhibited lower concentrations of several elements, suggesting a dilution effect rather than bioaccumulation. These findings provide valuable insights for environmental contamination monitoring in polar ecosystems.

Trophic transfer describes how HMs move through food webs, from primary producers to apex predators [83]. This process involves bioaccumulation at each level and biomagnification, where metal concentrations rise as they move up the food chain [50]. The persistence and poor excretion of some metals—especially methylmercury—contribute to their buildup in higher trophic levels [84].

Mercury, cadmium, and lead typically enter aquatic food webs via phytoplankton, which absorb dissolved metals from water [85,86]. These metals are then transferred to herbivores, such as zooplankton, which feed on the contaminated phytoplankton [87]. Zooplankton are then eaten by small fish and crustaceans, resulting in further metal accumulation up the food web [87]. Mercury (Hg) consistently biomagnifies, with its concentration increasing along the food chain, making apex predators particularly vulnerable [88]. Studies from the Mediterranean, South China Sea, and Amazon Coast show that top predators accumulate far more mercury than planktivorous or benthic feeders [27,69].

Other metals exhibit variable trophic transfer efficiency. Copper (Cu) and zinc (Zn) show strong bioaccumulation, particularly in carnivorous fish, due to their dietary exposure to contaminated prey [26,56]. Essential metals like Cu and Zn transfer more efficiently through food webs than toxic metals like lead (Pb) and cadmium (Cd), which biomagnify less due to lower bioavailability and faster excretion [44,54].

HMs movement through food webs illustrates the interconnected roles of producers, invertebrates, and predators in biomagnification [89].

Primary Producers: Phytoplankton play a foundational role by absorbing metals directly from the surrounding water. The high surface-to-volume ratio of these microscopic organisms allows them to efficiently sequester metals such as cadmium and mercury. Once incorporated into their biomass, these metals become bioavailable to higher trophic levels [89,90].

Herbivores and Zooplankton: Zooplankton, which graze on phytoplankton, act as key conduits for metal transfer in food webs [87]. Studies have shown that cadmium and mercury accumulate significantly in zooplankton tissues, binding to cellular structures. This stage is key to vertical transfer, as zooplankton are eaten by small fish and other consumers [87]. Research has indicated that biomagnification is most effective for mercury and essential trace metals such as Zn and Cu, while Pb and Cd remain largely confined to benthic organisms due to their limited dietary transfer [26,28].

Predatory Fish: At higher trophic levels, predatory fish such as tuna, mackerel, and swordfish accumulate metals from their prey [91]. Methylmercury, in particular, is highly biomagnified in these species due to its lipophilic nature and strong affinity for muscle proteins. Filice et al. (2023) [44] found that mercury and lead biomagnify in top predators, while cadmium and copper are more prevalent in benthic species, suggesting that some metals are more influenced by habitat exposure than trophic level [44].

Numerous studies provide robust evidence of trophic magnification of HMs in marine food webs, particularly for mercury, which accumulates most efficiently through dietary exposure [86,88]. In the North Atlantic, for example, mercury concentrations in tuna were found to be over 1000 times higher than those in primary producers [39]. Similarly, studies in Jiaozhou Bay found that methylmercury (MeHg) was more efficiently absorbed and retained than inorganic Hg (IHg), reinforcing its dominance in trophic magnification processes [40].

In contrast, cadmium and lead do not biomagnify as strongly but accumulate in benthic food webs, particularly in sediment-feeding organisms and their predators [44,54]. Lead concentrations are highest in benthic and demersal species, often accumulating in gills and bones rather than muscle tissues, limiting their biomagnification potential [82].

Some metals exhibit biodilution, with concentrations decreasing at higher trophic levels [92]. Arsenic (As), cadmium (Cd), and lead (Pb) exhibit this trend, particularly in the Amazon Coast and South China Sea, where lower trophic-level species accumulate higher concentrations than top predators [28,69]. This implies that these metals are either efficiently excreted or poorly assimilated at higher trophic levels, preventing their magnification within the food web [93].

Species-specific metabolic factors also influence metal retention. Some metals accumulate more efficiently in muscle tissues, while others are concentrated in liver and kidney tissues due to their detoxification roles [41]. Cesium (Cs), for example, biomagnifies in muscle tissue, whereas most other elements exhibit biodilution in gills, liver, and skin, likely due to differences in excretion rates [75].

Biomagnification effects are the most severe in top predators, which consume large amounts of contaminated prey. These impacts affect not only fish but also fish-eating birds, marine mammals, and humans [94].

Fish-Eating Birds: Species such as cormorants and pelicans, which rely heavily on contaminated fish, exhibit elevated levels of mercury and cadmium in their tissues [95]. These metals have been shown to impair reproductive success, leading to eggshell thinning and reduced hatchling viability [28].

Marine Mammals: Dolphins, seals, and other marine mammals are at significant risk due to their consumption of large predatory fish [96]. High mercury levels in these animals have been linked to neurological damage and immune suppression, making them particularly vulnerable to heavy metal toxicity [69].

Large Pelagic Fish: Apex predators such as tuna and swordfish suffer physiological disruptions from mercury accumulation, including reduced swimming efficiency and reproductive impairments [97]. These effects compromise their survival and have cascading impacts on the stability of marine ecosystems [27,39].

The trophic transfer and biomagnification of HMs highlight the interconnectedness of marine food webs and the cumulative risks posed by these persistent pollutants [20]. From primary producers to apex predators, the magnification of metals such as mercury and cadmium threatens marine biodiversity and ecosystem health, while posing significant risks to human consumers [98].

**Table 2:** Summary of studies on trophic transfer and biomagnification of heavy metals in marine ecosystems.

Reference	Region/HMs Studied	Key Findings
	Europe	
Pouil et al., 2016 [34]	Mediterranean Sea/turbot (*Scophthalmus maximus*)/Co, Mn, Zn	No biomagnification was observed, as metal uptake did not correlate with the trophic level. Instead, metal assimilation was influenced by prey tissue metal bioavailability, challenging conventional biomagnification models. These findings suggest that environmental and physiological factors have a greater impact on metal retention than the trophic position, offering a more nuanced understanding of metal transfer dynamics in marine food webs.
Zupo et al., 2019 [27]	Mediterranean Sea/Hg	Mercury concentrations increased along the food web, with apex predators exhibiting the highest levels due to biomagnification driven by prey consumption and trophic dynamics. These findings highlight the risks mercury poses to higher trophic-level species and underscore its relevance for human health and food web contamination assessments.
Vetsis et al., 2021 [75]	Thermaikos Gulf, Eastern Mediterranean Sea/28 demersal and pelagic fish species/27 metals	Cesium biomagnifies in muscle tissue, increasing with the trophic level due to efficient dietary absorption and retention. In contrast, most other elements exhibited biodilution across gills, liver, muscle, and skin, with concentrations decreasing at higher trophic levels. These patterns suggest that metabolic regulation and excretion limit the accumulation of many elements, while cesium persists in muscle, making it more susceptible to biomagnification. The findings highlight species-specific differences in metal metabolism and elimination within marine food webs.
Filice et al., 2023 [44]	Northwestern Mediterranean Sea/*Galeus melastomus*, *Conger conger*, *Mullus barbatus*, *Dentex dentex*, *Zeus faber*, *Sparus aurata*/Hg, Pb, Cd, Zn, Cu, Ni, Fe	Biomagnification of mercury and lead was observed, with the highest concentrations found in the top predator *Galeus melastomus*. In contrast, cadmium and copper were more prevalent in benthic species such as *Conger conger* and *Mullus barbatus*, suggesting that habitat exposure plays a greater role than the trophic level for certain metals. These findings highlight species-specific bioaccumulation patterns influenced by both feeding behavior and environmental conditions.
	Asia	
Qiu, 2015 [26]	Daya Bay, South China/carnivorous (*Saurida undosquamis*) and planktivorous (*Stromateoides argenteus*) fish/Cu, Zn, Pb, Cd	Bioaccumulation patterns varied by the species and trophic level. Carnivorous fish (*Saurida undosquamis*) accumulated higher levels of copper and zinc due to their top food web position, while planktivorous species (*Stromateoides argenteus*) showed moderate accumulation from lower trophic exposure. Lead and cadmium levels were generally low, but benthic organisms such as shrimp, crab, and shellfish exhibited higher cadmium BAFs due to sediment contact. Copper and zinc bioaccumulated and biomagnified efficiently, whereas lead and cadmium showed limited biomagnification due to lower bioavailability and faster excretion. These patterns highlight the importance of feeding habits and sediment exposure in metal transfer and risk to apex predators and human consumers.
Wang et al., 2019 [39]	China (marine and freshwater systems)/various fish species/mercury (Hg), methylmercury (MeHg)	Mercury bioaccumulation followed trophic patterns, with carnivorous fish exhibiting the highest levels, followed by omnivores and herbivores, reflecting biomagnification through the food chain. Predators accumulated more mercury through consumption of mercury-rich prey, while herbivores had minimal exposure. Methylmercury (MeHg) was more efficiently absorbed and retained than inorganic Hg, with the dietary intake, growth rate, and biotransformation influencing accumulation. Biokinetic modeling confirmed diet as the primary Hg source and showed that faster-growing fish had lower Hg concentrations due to growth dilution, while MeHg persisted in tissues, reinforcing its biomagnification across aquatic food webs.
Yang et al., 2020 [99]	South China Sea/*Sardinella albella*/As, Pb, Zn, Hg, Cu, Cd	Stable isotope analysis confirmed that *Sardinella albella* (trophic level 2.76) primarily feeds on zooplankton. No correlation was found between the trophic level and heavy metal concentrations, indicating that metal accumulation was driven more by environmental contamination than biomagnification. Muscle metal concentrations followed the order As > Pb > Zn > Hg > Cu > Cd, with most levels below pollution thresholds except for lead and arsenic, suggesting localized pollution sources. These findings emphasize the importance of environmental monitoring and the trophic context in evaluating metal accumulation in commercial fish species.
Mao et al., 2021 [40]	Jiaozhou Bay (China)/various aquatic organisms (fish, crustaceans, mollusks)/total mercury (THg), methylmercury (MeHg)	Total mercury (THg) and methylmercury (MeHg) concentrations were highest in fish, followed by crustaceans and mollusks. Mercury biomagnified in mollusks and fish but not in crustaceans, with MeHg showing greater bioaccumulation and trophic transfer efficiency than inorganic Hg. MeHg was effectively transferred through the food web, with notable uptake from suspended particulates. Lower trophic magnification factors (TMFs) compared to global averages were linked to overfishing and rapid growth rates, emphasizing the role of local environmental and biological factors in shaping mercury dynamics in urbanized coastal systems.
Huang et al., 2022 [56]	East China Sea/demersal and pelagic fish/As, Hg, Cd, Cu, Zn, Pb, Cr	Biomagnification of the essential metals zinc and copper was observed in apex predators such as *Muraenesox cinereus*, while toxic metals like cadmium and mercury showed lower biomagnification, likely due to limited assimilation and efficient excretion. Fatty acid profiles correlated with the metal levels, linking dietary habits to contamination patterns. These findings emphasize the role of trophic pathways and feeding behavior in metal transfer, with implications for food web contamination and ecological risk.
Javanshir Khoei, A., 2022 [57]	Oman Sea/7 pelagic and demersal fish species/Ni, Cr, Hg, Cd, Pb	The trophic level influenced metal accumulation in muscle tissue, with carnivorous and euryphagous species—both pelagic and demersal—accumulating higher metal concentrations than phytoplankton-feeding and herbivorous fish. These bioaccumulation patterns reflect trophic transfer dynamics and have important implications for seafood safety in commercially important fish species.
Yang et al., 2022 [69]	South China Sea/84 fish samples from various species collected from nearshore and offshore regions/Ni, Cu, As, Cd, Pb, Hg	Mercury biomagnified in both nearshore and offshore marine food webs, increasing with the trophic level, while copper biomagnified only in offshore fish, suggesting regional environmental or dietary influences. In contrast, nickel and lead showed biodilution, decreasing at higher trophic levels due to efficient excretion or metabolic regulation. Stable isotope analysis revealed distinct food web structures and dietary sources between regions, shaped by organic matter input and nutrient cycling. These findings offer insights into regional contamination patterns and trophic transfer dynamics.
	Africa	
Le Croizier et al., 2016 [54]	Dakar and Casamance, Senegal/fish and benthic prey/Cd, Pb, Fe, Zn, As, Ni, Sn, Co	Metal bioaccumulation in fish was strongly influenced by trophic interactions and feeding habits. In heavily impacted areas like Dakar, benthic prey consumption was the primary pathway for metal uptake, while fish from less polluted regions like Casamance, where pelagic prey dominated, exhibited lower metal levels. Fatty acid profiles proved more reliable than stable isotopes in linking diet to metal accumulation, underscoring the role of feeding ecology in food web contamination.
	America	
Ruelas-Inzunza et al., 2017 [79]	SE Gulf of California/white mullet (*Mugil curema*), striped mullet (*Mugil cephalus*)/total mercury (THg) and methylmercury (MeHg)	No strong evidence of mercury biomagnification was found in these mullets, as total mercury (THg) levels were comparable to those in similar species from other Mexican waters. However, methylmercury (MeHg) exposure was approximately three times higher than THg, indicating diet as the primary route of accumulation. Mercury levels were lower than those reported in mugilids from Argentina, India, and the Mediterranean, supporting the need for region-specific assessments in fisheries and food safety regulation.
Quintela et al., 2019 [42]	Taim wetlands, Southern Brazil/fish, reptiles/As, Pb	Arsenic concentrations were high across all species, with the highest levels found in the herbivorous–insectivorous *Astyanax aff. fasciatus* and detritivorous *Cyphocharax voga*, while lead concentrations were generally low, peaking in *Caiman latirostris*. Arsenic exhibited biodilution, decreasing with the trophic level, whereas lead showed no consistent accumulation pattern along the food chain. These findings provide insight into trophic transfer dynamics and highlight the influence of feeding habits on heavy metal distribution in wetland fish and reptiles.
Squadrone et al., 2020 [33]	Caribbean Sea/Indo-Pacific lionfish (*Pterois* spp.)/23 trace elements	No biomagnification was observed in lionfish, as metal concentrations did not increase with the trophic level. Instead, accumulation was driven by environmental exposure and organ-specific retention, with the liver and kidneys being the primary sites of metal storage. These findings reinforce the role of lionfish as a useful bioindicator of marine contamination, rather than a species affected by the significant trophic transfer of metals.
Figueiredo et al., 2020 [81]	Gulf of California (Guaymas Basin)/*T. mexicanus*, *B. panamense* (mesopelagic prey); predators: *Dosidicus gigas*, *Stenella longirostris*, *Physeter catodon*, *Eschrichtius robustus*/Cu, Zn, Cd, Pb	Biomagnification factors (BMFs) were >1 for all metals except Pb in *Dosidicus gigas*, indicating potential for trophic transfer to top predators. The study highlights the key role of myctophids as vectors of trace elements in the Gulf of California pelagic food web, especially in oxygen minimum zone ecosystems.
Johnson et al., 2021 [45]	Atlantic Coast (Florida, USA)/invasive lionfish (*Pterois volitans/miles*)/total mercury (THg)	Despite occupying a high trophic position, lionfish accumulated less mercury than comparable reef predators, likely due to species-specific physiological traits or rapid growth rates that limit Hg retention. Mercury levels remained below established safety thresholds, supporting the suitability of lionfish as a safe option for human consumption and a viable target for sustainable fisheries.
Serviere-Zaragoza et al., 2021 [80]	Gulf of California (Santa Rosalía and Bahía de La Paz)/*Kyphosus vaigiensis* (herbivore), *Stegastes rectifraenum* (omnivore), *Balistes polylepis* (carnivore)/Cd, Pb, Cu, Zn, Fe	Stable isotope analysis (δ^13^C and δ^15^N) was used to estimate the trophic positions (ranging from 2.0 to 3.3). The study found no consistent biomagnification patterns across metals. Zn showed increased concentrations in higher-trophic-level species, but Cd and Pb did not correlate with the trophic level. Feeding ecology and local environmental conditions were more influential than the trophic position alone in determining metal accumulation.
Souza-Araujo et al., 2022 [28]	Amazon Coast/47 cartilaginous and teleost fish species/As, Cd, Hg, Pb	Arsenic, cadmium, and lead concentrations decreased with an increasing trophic level, indicating trophic dilution, while mercury exhibited clear biomagnification, increasing in top predators. Lower trophic species, such as plankton and detritus feeders, accumulated higher levels of As, Cd, and Pb. These patterns underscore the influence of dietary habits and metabolism on metal accumulation, and highlight important considerations for ecotoxicology, seafood safety, and species-specific risk assessments in fisheries management and environmental monitoring.
Valladolid-Garnica et al., 2023 [100]	Pacific Coast (Southeastern Gulf of California)/multiple species (zooplankton, benthic invertebrates, and fish across trophic levels)/Cd, Cu, Mn, Pb, Zn	Investigated bioaccumulation and trophic transfer across the marine food web. Patterns varied by metal: some (e.g., Mn and Zn) showed biodilution, while others (Cd and Pb) had the potential for accumulation in higher trophic levels. Results emphasized species-specific differences and the role of the trophic position in HM exposure.
Szteren et al., 2023 [101]	Pacific Coast (Bahía Magdalena, Baja California Sur, Mexico)/multiple prey species; top predator: *Zalophus californianus* (California sea lion)/trace and toxic metals (including Hg)	Evaluated metal-specific biomagnification and trophic dilution within a benthic–pelagic coastal food web. Mercury exhibited clear biomagnification in higher trophic levels. The study provides baseline concentrations for several species and emphasizes the sea lion’s role as a bioindicator for trophic transfer of metals in coastal ecosystems.
	Antarctica	
Queiros et al., 2023 [82]	Southern Ocean/Antarctic toothfish (*Dissostichus mawsoni*)/27 trace elements	In *Dissostichus mawsoni*, no evidence of bioaccumulation or biomagnification was found for the studied elements, contrasting with earlier findings on mercury in this species. These results suggest that environmental factors, rather than trophic transfer, are the primary drivers of trace element concentrations, challenging previous assumptions about mercury dynamics in Antarctic food webs.

In conclusion, an integrated understanding of HM bioaccumulation in marine fish requires the consideration of both the physiological mechanisms of metal uptake and the environmental context of exposure. While this review highlights a wide range of species- and tissue-specific accumulation patterns, variability in bioconcentration factors (BCFs) and bioaccumulation factors (BAFs) further emphasizes the complex interplay of biological and ecological drivers. Evidence from both experimental and field-based studies highlights the tissue-specific nature of HM accumulation in fish, and the influence of the exposure route and species ecology. In a laboratory study, Sauliutė et al. (2017) [102] reported high bioconcentration factors (BCFs) in *Salmo salar*, with copper reaching up to 594 in liver and zinc reaching around 360 in gills, reflecting their roles in detoxification and direct waterborne uptake. Even non-essential metals such as lead (Pb) and cadmium (Cd) showed notable BCF variation across tissues and time, particularly in muscle and gills, likely influenced by physiological processes and metal interactions. Complementary field data from Olayinka-Olagunju et al. (2021) [103] revealed that pelagic species exhibited the highest bioaccumulation factors (BAFs) for zinc in gills, while benthic species showed greater accumulation of Pb and Mn, consistent with sediment exposure. The general tissue accumulation trend—gills > muscle > liver > heart—further underscores the gills as a key site of metal uptake in both laboratory and field conditions [102,103]. These findings reinforce the importance of integrating metal-specific, tissue-level accumulation data with the ecological context to improve bioaccumulation assessments and risk evaluation.

Aquatic organisms are primarily exposed to metals through the following two mechanisms: direct absorption from the abiotic environment, such as water and sediments, and indirect exposure through their prey or food sources. This dual pathway facilitates the accumulation of HMs within the food chain, leading to increased concentrations as they move up the trophic levels. The dominant uptake routes also vary according to species ecology and life history traits. Pelagic fish primarily accumulate metals through dietary exposure to plankton and small fish [87,104], whereas demersal fish face a more complex exposure regime that includes sediment ingestion, gill absorption from resuspended particles, and direct dermal contact with contaminated substrates [105]. Gills play a particularly important role in the absorption of waterborne metals such as copper (Cu) and zinc (Zn), especially in early life stages or in species with high metabolic and ventilation rates [106]. Meanwhile, the liver and kidneys function as key bioaccumulation sites for metals associated with detoxification and excretion, such as Cd and Pb, while muscle tissue becomes the primary repository for MeHg, with direct implications for seafood safety [107,108].

Several biological factors influence the efficiency and selectivity of metal accumulation in fish, including the species-specific physiology, size, age, sex, reproductive status, metabolic activity, and feeding behavior [86]. For example, active predators at higher trophic levels generally exhibit greater accumulation of mercury due to biomagnification [109], while benthic feeders ingest sediment-bound metals that may not efficiently transfer through food webs but accumulate locally [86]. Abiotic factors such as the water pH, salinity, temperature, and habitat characteristics (e.g., oxygenation and sediment type) also modulate metal bioavailability and speciation [110], further complicating predictive assessments. Importantly, dietary intake has been identified as the most influential pathway in long-term accumulation, particularly for non-essential and highly toxic metals such as Hg and Cd [111].

Despite progress in characterizing these patterns, critical knowledge gaps persist. There is a need for standardized comparative BCF data across species and habitats to improve cross-study integration and risk modeling [112,113]. Moreover, while bioaccumulation from water and diet is relatively well documented, the role of sediment as a long-term source and modulator of metal uptake remains underexplored, especially in field conditions [114]. Further research should also prioritize integrative approaches that combine metal speciation, biomarker responses, and sub-organismal effects with bioaccumulation data to better predict ecological risks and inform fisheries management. Understanding these interactions at multiple scales is essential to accurately assess the trophic transfer potential of metals and the long-term implications for ecosystem and human health.

## 4. Ecological Consequences of Heavy Metal Pollution in Marine Ecosystems

HM pollution affects all levels of marine life—from individual organisms to entire ecosystems [115]. Such disruptions alter trophic interactions, population dynamics, and community structure, threatening ecosystem services, fisheries, and public health [116].

### 4.1. Population-Level Effects

HM contamination disrupts fish reproduction and development by interfering with hormones, gonadal function, and embryogenesis [117,118]. Metals such as cadmium (Cd), chromium (Cr), copper (Cu), mercury (Hg), lead (Pb), nickel (Ni), and zinc (Zn) have been documented to induce gonadal abnormalities, disrupted hormone levels, genetic alterations, and impaired reproductive success in various fish species [119]. Such effects can lead to population declines, biodiversity loss, and disrupted food webs [118].

HMs interfere with the hypothalamic–pituitary–gonadal (HPG) axis, causing hormonal imbalances and structural gonadal damage [120,121]. Cadmium, for example, mimics calcium ions and disrupts the HPG axis by misactivating gonadotropin-releasing hormone (GnRH) signaling pathways. This misregulation affects luteinizing hormone (LH) and follicle-stimulating hormone (FSH) synthesis in the pituitary, ultimately impairing gonadal steroidogenesis and gametogenesis [121]. Studies report altered expressions of key genes such as *gnrh1*, *lhβ*, *fshβ*, *ar*, and *cyp19a1a* in fish brains and gonads following exposure to cadmium, copper, and mercury [122]. These effects often reduce estradiol (E2) and testosterone (T) levels, disrupting key hormonal feedback loops [122].

Cadmium (Cd) has been shown to suppress spermatogenesis, cause testicular necrosis, Sertoli cell hypertrophy, liver nuclear hypertrophy, and bile stagnation, and induce low plasma cortisol and thyroxine (T4) levels, all indicative of endocrine disruption [49,123]. Cr, Cu, Hg, and Ni affect the gonadal structure, disrupt steroid hormone synthesis, and alter adrenal and reproductive gene function [119,124]. Mercury (Hg) is particularly toxic to reproductive health, leading to ovarian degeneration, impaired ovarian recrudescence, the degeneration of hypothalamic neurons, and the inhibition of gonadotropin-secreting cells [125,126]. Lead (Pb) exposure has been linked to thyroid dysfunction, increased necrosis in gonadal tissue, liver cell damage, mitochondrial autophagy, altered immunity, and Sertoli cell hypertrophy [119]. Studies on zebrafish (*Danio rerio*) demonstrated that exposure to copper (10–40 μg/L) caused gonadal dysfunction, reduced body weight, altered steroid hormone levels, and disrupted reproductive gene expression in both the brain and gonads [124].

In pejerrey fish (*Odontesthes bonariensis*), exposure to a mixture of Cd, Cr, Cu, Ni, Pb, and Zn resulted in a significant increase in gonadotropin-releasing hormone expression, while gonads exhibited fibrosis, sperm lobule shortening, and cellular pyknotic changes, indicating long-term reproductive damage [119]. In Mozambique tilapia (*Oreochromis mossambicus*), cadmium exposure triggered early sexual maturation in juveniles but caused gonadal degeneration in adults [127].

HM exposure severely affects fish embryogenesis, larval growth, and skeletal formation [128]. Methylmercury and lead delay hatching and cause craniofacial deformities, spinal and heart defects, and neurotoxicity [125,129]. Studies on rainbow trout (*Oncorhynchus mykiss*) showed that cadmium exposure at 0.05–2.50 μg/L resulted in premature hatching at low concentrations but delayed hatching at higher concentrations. Exposed larvae were significantly shorter, and plasma sex steroid levels were abnormally elevated, indicating hormonal dysregulation [129]. Similarly, Japanese medaka (*Oryzias javanicus*) exposed to cadmium (280 μg/L) exhibited differential gene expression in the liver, reflecting major metabolic and physiological disturbances [49]. Field studies have confirmed these laboratory findings. European perch (*Perca fluviatilis*) embryos from metal-contaminated sites exhibited spinal deformities and reduced hatching success, while brown trout (*Salmo trutta*) populations in Pb-contaminated rivers displayed growth retardation and cranial asymmetry [130].

With the increased use of metal nanoparticles (NPs), their accumulation in aquatic environments has raised concerns regarding their bioavailability and reproductive toxicity. Copper nanoparticles (Cu-NPs) at 20 and 100 μg/L caused oxidative stress, liver lesions, and structural disruptions in the gills and kidneys of common carp (*Cyprinus carpio*) [131]. Zinc oxide nanoparticle (ZnO-NP) exposure resulted in testicular atrophy, reduced spermatogenesis, and oxidative stress [132]. Exposure to bisphenol A (BPA) and titanium dioxide nanoparticles (n-TiO_2_) in zebrafish led to reduced thyroxine (T4) levels in adults, impaired maternal hormone transfer, and developmental neurotoxicity in larvae [133]. Such interactions between endocrine disruptors (EDs) and nanoparticles complicate metal toxicity and raise concerns regarding co-exposure risks in contaminated environments [134].

HMs modulate sex steroid levels, alter gene expression in reproductive organs, and disrupt neuroendocrine pathways [135]. Copper (Cu) and lead (Pb) exposure in zebrafish resulted in growth suppression, gonadal abnormalities, and altered E2 and T hormone levels [124]. Northern pike (*Esox lucius*) and yellow perch (*Perca flavescens*) exposed to PAHs, PCBs, and HMs exhibited chronic stress responses and altered adrenal function, further supporting the role of contaminants in neuroendocrine disruption [130]. Overall, heavy metal pollution has severe implications for fish reproductive health, embryonic development, and endocrine function, with metals such as cadmium, mercury, lead, and copper being the most toxic [118]. These metals disrupt hormonal balance, gonadal integrity, gene expression, and neurodevelopment, leading to fertility reduction, increased mortality, and population declines [136].

These reproductive and developmental impairments translate into broader ecological consequences by altering population dynamics over time. Chronic exposure to heavy metals reduces reproductive output, juvenile survival, and recruitment success, leading to smaller population sizes and shifts in population structure [117]. For instance, delayed sexual maturation, skewed sex ratios, and the intergenerational transfer of contaminants can reduce the genetic diversity and adaptive capacity [137]. Such population-level changes affect not only the resilience of affected fish species but also the stability of the ecosystems they inhabit [20]. Moreover, reduced prey availability due to population declines in lower trophic levels may exert bottom-up pressure on predatory species, compounding ecosystem-level disruptions [94].

Recent integrative modeling studies have highlighted how cumulative environmental pressures, including HM contamination, affect fish at the individual level in ways that scale up to population-level consequences [138]. For example, a study on gilthead seabream (*Sparus aurata*) in Mediterranean coastal lagoons used Partial Least Squares Path Modeling (PLS-PM) to integrate 10 environmental and 36 physiological variables into a multifactorial framework assessing the stress response. The model explained 54% of the variance in fish stress levels, with the inorganic contaminant load emerging as a significant predictor, alongside the lagoon features, trophic condition, and somatic structure. These findings underscore how pollutant exposure—especially from inorganic metals—interacts with other environmental and biological factors to influence physiological stress, with potential implications for reproductive output, growth, and long-term population viability [138].

### 4.2. Community-Level Effects

The impact of heavy metal pollution on marine biodiversity is significant [128]. Studies show that HMs impair reproduction, growth, and survival across a wide range of marine species—from microbes to mammals [139]. The degradation of critical habitats, such as coral reefs and mangroves, further exacerbates the loss of biodiversity, as these ecosystems are essential for many marine species [139,140]. For instance, HMs reduce species richness and evenness, shifting the community composition, and lead to the emergence of pollution-tolerant species [141,142]. This shift can destabilize ecosystems, as the loss of sensitive species disrupts ecological interactions and functions [143].

The community structure of marine ecosystems is undergoing significant changes due to heavy metal pollution, since it often leads to shifts in the community composition, as pollution-tolerant species outcompete sensitive ones [144]. For instance, mollusks and crustaceans that can bioaccumulate metals without significant physiological stress may dominate in heavily polluted areas, replacing more sensitive fish or invertebrate species [141,145]. This imbalance reduces biodiversity and functional redundancy—key elements of resilient ecosystems [146]. As sensitive species decline, more resilient, pollution-tolerant species often proliferate, leading to altered food webs and ecosystem dynamics [141,145]. This phenomenon is particularly concerning, as it can diminish the overall health and resilience of marine ecosystems, making them more vulnerable to additional stressors such as climate change and overfishing [140]. Such community shifts can impair ecosystem services like fisheries and coastal protection, which are vital for human livelihoods [145]. Thus, heavy metal pollution is a critical factor driving biodiversity shifts and changes in community structure within marine ecosystems [147]. The emergence of pollution-tolerant species and the decline of sensitive species highlight the need for comprehensive monitoring and management strategies to mitigate the impacts of pollution and preserve marine biodiversity [148].

Furthermore, HMs weaken prey populations by impairing their health and reducing their abundance [94]. Predators relying on this prey suffer too, disrupting the energy flow across the ecosystem [149]. Studies have shown that mercury-exposed small pelagic fish exhibit reduced swimming efficiency and escape responses, making them more vulnerable to predation and reducing their population size [150]. Such disruptions can trigger trophic cascades and destabilize marine food webs [151]. Heavy metal exposure has been shown to alter the feeding behavior of fish, making them more vulnerable to predation and further impacting predator–prey dynamics within these ecosystems [152,153]. The disruption of predator–prey interactions in marine ecosystems can be exacerbated by marine pollution [154]. Changes in the behavior and health of prey species can lead to altered predation patterns, which may have cascading effects throughout the food web [155,156]. For example, the presence of pollutants can impair the ability of prey to detect and evade predators, thereby increasing their susceptibility to predation [157]. Additionally, the decline of top predators due to overfishing and environmental stressors can lead to an overabundance of certain prey species, further destabilizing ecosystem dynamics [155,158].

The recovery of predator populations in marine reserves has been shown to influence prey dynamics significantly [159]. As predator numbers increase, the predation pressure on prey species can lead to shifts in their population dynamics and distribution [158]. This interplay between predator recovery and prey population dynamics highlights the importance of maintaining healthy predator populations to ensure the stability of marine ecosystems [160]. Overall, heavy metal pollution could severely impact marine ecosystems by disrupting predator–prey dynamics and threatening biodiversity. HM buildup harms marine life and alters the complex relationships that define food webs [20].

Overall, HM pollution exerts profound and multifaceted effects on the marine community structure. Beyond isolated species responses, these pollutants trigger cascading shifts that reshape biodiversity patterns, trophic interactions, and ecological functions [144,147]. The emergence of pollution-tolerant taxa and the decline of sensitive species reflect both ecological filtering and functional simplification within impacted habitats [161]. Such community-level transformations reduce ecosystem resilience, impair recovery potential, and threaten ecosystem services. Despite growing evidence, long-term studies on how chronic exposure alters species interactions and food web dynamics remain limited [20,86]. Future research should integrate ecological modeling and time-series biodiversity assessments to better understand these shifting baselines and inform adaptive management strategies [162].

### 4.3. Ecosystem-Level Impacts

HM pollution threatens marine ecosystems, particularly through sediment toxicity and altered bioturbation processes [163]. Persistent pollutants such as cadmium, copper, and mercury accumulate in marine sediments, leading to toxic effects on benthic organisms and disrupting trophic interactions within ecosystems [164,165]. These metals often originate from human activities—such as industrial discharge and urban runoff—which degrade water and sediment quality [166].

Bioturbation—the sediment-reworking activity of benthic organisms—modulates HM bioavailability in polluted environments. Depending on the species and sediment conditions, bioturbation can either bury metals deeper, reducing their bioavailability, or resuspend them into the water column [167]. Species such as amphipods and bivalves alter metal speciation and mobility by transporting particles, oxygenating sediments, and shifting redox conditions [168,169]. The activity of bioturbators can change the sediment chemistry, potentially increasing the bioavailability of toxic metals to other organisms [168]. Conversely, bioturbation may also promote sediment detoxification by enhancing microbial activity involved in the biogeochemical cycling of nutrients and contaminants [170]. However, high HM concentrations can impair the behavior and physiology of bioturbators, disrupting their ecological roles and altering sediment dynamics [167,171].

The impacts of HM pollution extend beyond individual species, affecting entire ecosystems and leading to trophic disruptions [172]. The presence of HMs can shift the community composition by favoring metal-tolerant species while excluding sensitive ones, thus disturbing the balance of ecological interactions [173]. Such shifts can have cascading effects on higher trophic levels, including fish populations that rely on benthic organisms for food [174]. Studies have shown that metal-induced sediment toxicity reduces the abundance and diversity of benthic communities, weakens ecological resilience, and impairs key ecosystem functions such as nutrient cycling and organic matter breakdown [175]. Studies also show that metal-contaminated sediments hinder the recruitment of sensitive species, promote pollution-tolerant taxa, and reshape trophic interactions in benthic communities [176,177].

Fisheries sustainability is increasingly threatened by the combined pressures of HM pollution and the resulting ecological changes [5,20]. Contaminated sediments can lead to fish population declines due to reduced food availability and increased toxicity within the food web [173]. Moreover, HM bioaccumulation in fish poses risks to human health, as these metals enter the food chain and impact seafood safety [166]. Effective management strategies are essential to mitigate the effects of HM pollution on marine ecosystems and ensure the sustainability of fisheries [178]. These strategies should include monitoring sediment quality, assessing the ecological consequences of bioturbation, and implementing pollution control measures to reduce HM inputs into marine environments [179,180]. Marine pollution significantly impacts ecosystem health through sediment toxicity, bioturbation processes, and trophic disruptions [181,182]. The interplay among these factors impacts benthic communities and poses risks to fisheries, calling for integrated and comprehensive management approaches [183].

HM contamination also affects the plankton community structure, leading to cascading impacts throughout the food web and undermining ecosystem stability [184]. Plankton form the foundation of marine ecosystems, and shifts in their composition can disrupt nutrient cycling and energy transfer to higher trophic levels, including fish populations [185,186]. HMs such as cadmium and mercury are toxic even at low concentrations, thereby reducing the growth and reproductive success in planktonic organisms [186]. This decline in plankton health can, in turn, affect fish species that depend on plankton as a primary food source [187,188].

Furthermore, the bioaccumulation and biomagnification of HMs along the food chain amplify ecological risks. As smaller organisms such as plankton accumulate metals, these contaminants are transferred to larger predators, including commercially important fish species [14]. This not only endangers marine life but also threatens human health through seafood consumption [188,189]. The implications for fisheries are significant, as declining fish stocks from heavy metal toxicity jeopardize the livelihoods of communities dependent on fishing [190,191].

Beyond altering plankton composition and trophic interactions [192], HM pollution can cause broader ecosystem-level changes. The presence of HMs can affect the physicochemical properties of marine environments, such as the pH and salinity, which are crucial for maintaining aquatic health [191]. Additionally, the persistence of HMs in sediments means that their effects can last long after pollution has ceased, thus continuing to affect marine organisms and ecosystem integrity [193]. The combined influence of HM pollution, plankton shifts, and ecological degradation highlights the need for robust environmental management. These changes weaken benthic–pelagic coupling and reduce the capacity of marine ecosystems to buffer environmental stress and recover from disturbances. Mitigation should focus on pollution reduction, the long-term monitoring of HM levels, and sustainable fishing practices to protect marine ecosystems and the coastal communities that rely on them [180,190,194].

In summary, the ecosystem-level consequences of HM pollution reflect the cumulative effects of physiological stress, species-level vulnerabilities, and altered community structures [128]. The persistent accumulation of metals in sediments—combined with bioturbation and trophic disruptions—leads to diminished ecological integrity and impaired ecosystem functioning [195]. These changes reduce biodiversity and threaten the productivity and sustainability of marine fisheries. However, predicting ecosystem responses remains challenging due to the complex interactions among multiple stressors, including eutrophication, climate change, and overfishing [196]. Future research should prioritize integrative, multi-trophic assessments and long-term ecological monitoring to better capture these synergistic effects and support ecosystem-based management.

### 4.4. Economic and Social Implications

The ecological consequences of heavy metal pollution are pervasive, spanning population-level disruptions [119], community composition shifts [141], and ecosystem service degradation [164]. The cascading effects of bioaccumulation and biomagnification affect both human health and economic stability, highlighting the need for comprehensive monitoring, mitigation, and regulation. These challenges highlight the deep connection between marine ecosystems and human societies, reinforcing the importance of a holistic approach to heavy metal pollution [180,194].

HM pollution poses major challenges to fishery sustainability, seafood safety, and the well-being of communities that rely on these resources [197]. The bioaccumulation of HMs, such as lead (Pb), cadmium (Cd), and mercury (Hg), in marine organisms can lead to severe health risks for both marine life and humans who consume contaminated seafood [51,89]. Studies show that HMs can accumulate in fish tissues at levels exceeding safety thresholds for human consumption, posing public health risks [152,198,199]. Industrial activities contribute significantly to the presence of HMs in coastal waters, which, in turn, affects the fish populations that inhabit these areas [152]. Similarly, research conducted in Bima Bay indicated that cadmium levels in milkfish exceeded established safety limits, underscoring the need for regulatory measures to mitigate pollution from community and industrial sources [200]. The consequences of such contamination extend beyond ecological impacts, directly affecting seafood safety and market viability, potentially leading to economic losses for fisheries and associated industries [201,202].

The economic impacts of heavy metal pollution are diverse and far-reaching [203]. They include the loss of market access for contaminated seafood, increased health care costs due to pollution-related illnesses, and the potential decline in tourism in affected coastal areas [204,205]. HMs reduce marine product quality and undermine consumer confidence, leading to reduced demand and economic downturns for local fisheries [199,206]. Furthermore, the social costs are significant, particularly for communities that rely heavily on fishing as a primary source of income [207]. The decline in fish populations due to pollution can lead to food insecurity and increased poverty levels among fishing communities [198,208].

The health risks of consuming contaminated fish are well documented [209]. HMs can cause a range of health issues, including neurological disorders, reproductive problems, and other chronic conditions [152,189,202]. The accumulation of these metals in the human body can lead to serious long-term health effects, necessitating the stringent monitoring and regulation of seafood products [198,199]. Additionally, the potential for HMs to biomagnify through the food chain raises concerns about the safety of consuming larger predatory fish, which often contain higher concentrations of toxins [91,210].

The widespread issue of HM pollution presents major challenges to fisheries, seafood safety, and the social and economic stability of coastal communities [147]. The economic consequences of heavy metal pollution are profound. Contaminated fisheries lead to reduced market demand, affecting local and global economies reliant on seafood exports [211]. The loss of consumer trust in seafood safety threatens industries, leading to costly mitigation efforts, while fishing communities face economic and food security risks [212]. Tackling these challenges requires pollution reduction, seafood safety monitoring, and community engagement to ensure sustainable marine resources [211].

Overall, the ecological impacts of HM pollution in marine ecosystems span multiple biological levels, from molecular disruptions in individual organisms to large-scale alterations in the community structure and ecosystem functioning [147]. Numerous studies have shown that metal-induced reproductive toxicity, endocrine disruption, developmental abnormalities, and immunosuppression contribute significantly to reduced fitness and population declines in affected fish species [19,21]. Such effects are especially pronounced in species inhabiting metal-contaminated nursery grounds or coastal zones with high sediment-bound contamination [147,213]. At the community level, the selective pressure imposed by chronic exposure fosters shifts in species composition, favoring pollution-tolerant organisms at the expense of sensitive taxa. Such changes can reduce biodiversity, weaken resilience, and simplify food webs, diminishing their functional redundancy [144,214].

At the ecosystem scale, the persistence of HMs in sediments and their interaction with bioturbation processes alters nutrient cycling and trophic dynamics, with cascading effects on predator–prey relationships and energy flow [215,216]. These disruptions are further amplified when sublethal physiological impairments in prey species—such as impaired swimming performance or predator evasion—translate into broader population-level effects [21,217]. The combined impact of HM pollution and other stressors—such as climate change, acidification, and overfishing—is still poorly understood, but likely plays a crucial role in shaping the trajectory of ecosystem degradation [218,219].

Despite a growing body of evidence documenting various effects, important knowledge gaps persist. Long-term field studies are needed to better quantify how chronic exposure to HMs influences recruitment success, food web stability, and ecological thresholds [220]. Additionally, there is a lack of integrative assessments that connect biochemical biomarkers with population-level and ecosystem-scale consequences [221,222]. Research combining sediment geochemistry, species-specific sensitivity, and bioenergetic modeling could offer predictive insights into future ecosystem responses [223]. Finally, more attention must be directed toward evaluating the effectiveness of pollution mitigation strategies in restoring ecological balance, particularly in coastal regions where anthropogenic pressures are most intense.

## 5. Conclusions

This review emphasizes that a comprehensive, multi-level understanding of HM pollution in marine fish is essential for effective risk assessment, monitoring, and ecosystem-based management. Species ecology, life history traits, habitat conditions, and regional pollution sources all play critical roles in shaping both the bioaccumulation pathways and the ecological consequences of HMs. Based on recent experimental and field studies, several key patterns emerge.

The highest HM concentrations were generally reported in demersal species inhabiting coastal and estuarine zones, where sediment-bound metals and anthropogenic inputs are elevated. The most frequently studied species include commercially and ecologically important taxa such as gilthead seabream (*Sparus aurata*), red mullet (*Mullus barbatus*), European seabass (*Dicentrarchus labrax*), anchovy (*Engraulis encrasicolus*), whiting (*Merlangius merlangus*), and horse mackerel (*Trachurus* spp.). These species are widely distributed and commonly targeted in biomonitoring programs across regions such as the Mediterranean, Black Sea, and South and East Asia due to their diverse ecological niches, varying trophic levels, and relevance for seafood safety.

Methylmercury is consistently the most concerning metal for human health due to its strong biomagnification in the muscle tissues of top predators (e.g., shark, tuna, and swordfish), while cadmium and lead accumulate primarily in the liver and kidneys, indicating chronic exposure and detoxification burdens. Thus, species posing elevated dietary risks include tuna, shark, swordfish, and red mullet due to either their trophic position or habitat-linked exposure. These species often show high concentrations of HMs, with implications for seafood safety and consumer health.

At the population level, chronic HM exposure is often linked to reproductive impairment, delayed maturation, reduced offspring viability, and altered sex ratios, all of which threaten recruitment and long-term population stability. At the community and ecosystem levels, HM contamination drives shifts in species composition, favoring pollution-tolerant taxa and reducing the overall biodiversity and food web complexity. These changes weaken ecosystem resilience and can amplify the effects of other stressors such as overfishing or climate change.

This review consolidates the current understanding of HM bioaccumulation and trophic transfer in marine fish, alongside their ecological and ecosystem-level impacts. The accumulation of metals in specific tissues—the liver, kidney, gill, and muscle—is influenced by the metal type, fish species, habitat use, and exposure routes. Methylmercury in muscle tissue and cadmium in the liver and kidney consistently emerge as the most concerning patterns due to their persistence, toxicity, and relevance to seafood safety. At higher ecological levels, bioaccumulation and biomagnification contribute to reproductive impairments, behavioral alterations, and declining fish populations, which, in turn, destabilize food webs and reduce marine biodiversity. Shifts toward pollution-tolerant species and disrupted predator–prey dynamics reflect the systemic nature of metal-induced ecological degradation. This evidence shows that HM contamination is not just a localized toxic issue, but a widespread ecological stressor, with long-lasting consequences for marine ecosystem function and resilience.

HM pollution in marine ecosystems presents a critical environmental challenge with far-reaching consequences for biodiversity, ecosystem services, and human health [5]. As HMs accumulate in marine organisms and transfer through food webs, they disrupt the ecological balance, impair nutrient cycling, and diminish biodiversity [224]. These effects are particularly concerning for marine fish, which serve as key components of marine ecosystems and vital food sources for human populations [225]. The persistence of HMs in sediments further exacerbates contamination, prolonging risks even when external inputs are reduced [226]. HM pollution has major economic and social consequences, reducing seafood market demand, harming global trade, and threatening fishing-dependent livelihoods [227]. It erodes consumer trust, leading to costly mitigation efforts, while vulnerable populations face heightened health risks from contaminated fish [227].

Addressing these challenges requires a multidisciplinary approach. Advances in molecular biology, nanotechnology, and ecosystem-based management offer promising tools for monitoring and mitigating HM contamination [228]. Long-term monitoring programs, enhanced with remote sensing and data analytics, are essential for identifying pollution trends and assessing the efficacy of regulatory measures [229]. Future research should examine the interactions between HMs and emerging pollutants, including microplastics and pharmaceuticals, as well as the influence of climate change on heavy metal bioavailability and toxicity [180,230].

Policy interventions play a crucial role in mitigating heavy metal pollution. Strengthening international regulations, enforcing stricter controls on industrial emissions and agricultural runoff, and integrating pollution management into marine conservation strategies are fundamental steps [231]. Public awareness campaigns should complement these efforts by educating consumers on seafood safety and advocating for informed dietary choices, particularly for high-risk populations [232,233]. Addressing HM pollution is crucial due to its impact on both marine ecosystems and human societies. Bridging knowledge gaps, promoting research collaboration, and enforcing effective policies can help mitigate its effects. A collective commitment to sustainability and regulation is essential to protect biodiversity, maintain ecosystem functions, and safeguard public health [147,234].

## 6. Future Directions

Although HM contamination is well documented, several critical knowledge gaps remain. Future studies should prioritize long-term field-based monitoring to assess the cumulative and chronic impacts on fish populations, particularly in metal-polluted nursery grounds and coastal habitats. More integrated research is needed to link sub-organismal biomarkers (e.g., oxidative stress, endocrine disruption, and DNA damage) with ecological outcomes, like reduced fitness, population declines, or food web disruptions. More attention should be given to the role of sediments as both sinks and secondary sources of metal exposure, particularly in demersal fish. Interdisciplinary approaches combining toxicology, ecology, fisheries science, and policy are essential for evaluating the effectiveness of mitigation strategies and ensure the long-term sustainability of marine resources under increasing anthropogenic pressure.

Despite progress in understanding HM bioaccumulation and trophic transfer in marine fish, several key gaps remain that warrant further investigation. Future research should focus on the following key areas:

### 6.1. Species-Specific and Regional Variability

Current studies show inconsistent bioaccumulation patterns across different fish species and marine environments. Future research should explore how species physiology, feeding habits, and habitat conditions influence heavy metal uptake and retention [27,56]. Longitudinal studies should be conducted across multiple marine ecosystems to identify spatiotemporal trends in metal contamination and assess how climate change (e.g., temperature shifts and ocean acidification) may affect bioaccumulation dynamics [54].

### 6.2. Advancing Trophic Transfer Studies

While the biomagnification potential of metals like mercury is well-documented, other metals (e.g., cadmium and lead) show inconsistent trophic transfer patterns. Future research should apply stable isotope analysis and advanced bioaccumulation models to distinguish dietary metal uptake from environmental exposure [28,40,44]. More studies should focus on lower trophic levels (phytoplankton, zooplankton, and benthic organisms) to refine our understanding of how metals enter and propagate through marine food webs [44].

### 6.3. Long-Term Monitoring and Risk Assessment

Most existing studies rely on short-term data. Establishing long-term monitoring programs is essential to track the cumulative effects of heavy metal pollution on fish populations and marine biodiversity [36,37,45,75]. Future studies should use multi-biomarker approaches to assess the sublethal effects of chronic HM exposure, such as oxidative stress, neurotoxicity, and endocrine disruption in fish [235].

### 6.4. Human Health Implications and Seafood Safety

Given the increasing reliance on seafood as a global protein source, there is a need to refine human health risk assessment models for heavy metal exposure through fish consumption [29,73]. Future studies should integrate consumption pattern data and metal bioavailability analyses to better estimate the risks associated with contaminated seafood [197].

### 6.5. Mitigation Strategies and Policy Development

Although pollution control efforts are in place, more research is needed on bioremediation and ecological restoration (e.g., phytoremediation and microbial detoxification) to mitigate heavy metal contamination in marine environments [47]. Strengthening international policies on industrial waste discharge, coastal management, and fisheries monitoring will be crucial in reducing metal inputs into marine ecosystems [231].

Addressing these research gaps will enhance our understanding of HMs pollution’s ecological and ecosystem-level impacts. Future research integrating multidisciplinary methods, advanced analytics, and long-term monitoring will be essential for protecting marine biodiversity, protecting seafood consumers, and informing sustainable environmental policies.

## Data Availability

Not applicable.
